# *O*-GlcNAc: A Bittersweet Switch in Liver

**DOI:** 10.3389/fendo.2014.00221

**Published:** 2014-12-17

**Authors:** Kaisi Zhang, Ruonan Yin, Xiaoyong Yang

**Affiliations:** ^1^Program in Integrative Cell Signaling and Neurobiology of Metabolism, Yale University School of Medicine, New Haven, CT, USA; ^2^Section of Comparative Medicine, Yale University School of Medicine, New Haven, CT, USA; ^3^Department of Cellular and Molecular Physiology, Yale University School of Medicine, New Haven, CT, USA; ^4^Department of Cell Biology, Yale University School of Medicine, New Haven, CT, USA

**Keywords:** *O*-GlcNAc, insulin, glucagon, liver metabolism, insulin resistance, NAFLD, liver fibrosis

## Abstract

The liver is a vital organ responsible for maintaining nutrient homeostasis. After a meal, insulin stimulates glycogen and lipid synthesis in the liver; in the fasted state, glucagon induces gluconeogenesis and ketogenesis, which produce glucose and ketone bodies for other tissues to use as energy sources. These metabolic changes involve spatiotemporally co-ordinated signaling cascades. *O*-linked β-*N*-acetylglucosamine (*O*-GlcNAc) modification has been recognized as a nutrient sensor and regulatory molecular switch. This review highlights mechanistic insights into spatiotemporal regulation of liver metabolism by *O*-GlcNAc modification and discusses its pathophysiological implications in insulin resistance, non-alcoholic fatty liver disease, and fibrosis.

## Introduction

The liver is the second largest organ and accounts for about 2% of the body mass of an adult human being. It is a major metabolic organ responsible for maintaining whole-body homeostasis in a changing nutritional environment. Dysregulation of liver metabolism is associated with a wide range of chronic liver disorders.

### Liver metabolism during the feeding/fasting cycle

The liver plays a key role in maintaining normal glucose levels between meals. When blood glucose is in excess (e.g., after a meal), the liver rapidly takes up glucose to produce glycogen (glycogenesis). When blood glucose levels fall below a normal range (72–85 mg/dL for healthy individuals), glycogen is broken down into glucose (glycogenolysis), which is then exported to the bloodstream. If the glycogen reserve is exhausted, the liver will generate glucose from non-carbohydrate carbon substrates, such as lactate, pyruvate, glycerol, and glucogenic amino acids (gluconeogenesis).

The liver also oxidizes triglycerides to produce energy during fasting. When carbohydrates and proteins are in excess, they are converted into fatty acids and triglycerides in the liver, and these are then exported and stored in adipose tissue. The liver is also responsible for producing lipoproteins, cholesterol, and phospholipids.

The metabolic function of the liver during the feeding/fasting cycle is tightly regulated by several endocrine hormones, particularly insulin and glucagon. Insulin enhances glucose uptake in muscle and adipose tissue and inhibits glucose production in the liver, thereby regulating blood glucose concentration. Insulin also stimulates the synthesis of fatty acids, glycogen, and proteins. The opposing effects are largely mediated by glucagon – it raises blood glucose concentration by promoting glycogenolysis and gluconeogenesis. These signaling and biochemical pathways can be regulated by insulin and glucagon at the transcriptional, translational, and post-translational levels.

### Insulin resistance and NAFLD

Since the liver is a vital organ to sustain metabolic homeostasis, chronic liver disorders are among the most devastating human diseases. Liver dysfunction can be a consequence of infection, immune disorders, alcohol- or drug-induced liver damage. It can contribute to a constellation of common metabolic disorders, including insulin resistance, non-alcoholic fatty liver disease (NAFLD), and non-alcoholic steatohepatitis (NASH).

Hepatic insulin resistance causes impaired glycogen synthesis and failure to suppress glucose production, eventually resulting in hyperglycemia. Insulin resistance is often associated with increased lipogenesis and hepatic steatosis (fatty liver).

Non-alcoholic fatty liver disease is now the most common liver disorder in the US, where 30% of the general adult population suffers from the disease (1). NAFLD can be characterized by excessive lipid accumulation in hepatocytes. In addition, a strong correlation between NAFLD and type 2 diabetes (T2D) has been reported: 70–80% of T2D and obesity patients have NAFLD and most patients with NAFLD have hepatic insulin resistance (2).

The majority of patients with NAFLD starts with simple steatosis and is often asymptomatic. However, a subset of NAFLD patients with simple steatosis can progress to NASH with manifestations of inflammation, hepatocellular injury, and fibrosis (3). Patients with fibrosis tend to have poor prognosis, often progressing to cirrhosis and even hepatocellular cancer (4). However, the molecular basis for the development of NAFLD and NASH is not clearly understood.

### *O*-GlcNAc modification

*O*-GlcNAcylation has emerged as an important regulatory mechanism underlying normal liver physiology and liver diseases. This post-translational modification uses UDP-GlcNAc as the substrate for the attachment of the acetylglucosamine (GlcNAc) moiety to the hydroxyl groups of serine and threonine residues of proteins (5, 6). Since UDP-GlcNAc is synthesized via the hexosamine biosynthesis pathway (HBP), which involves nutrients such as glucose, free fatty acids, uridine, and glutamine, *O*-GlcNAcylation can serve as a nutrient sensor, tuning various cellular processes in response to systemic metabolic status (7–9). The cycling of *O*-GlcNAcylation is controlled by two highly conserved enzymes: *O*-GlcNAc transferase (OGT) catalyzes the addition of *O*-GlcNAc to proteins and *O*-GlcNAcase (OGA) catalyzes the removal of the monosaccharide (10–12). This dynamic modification is prevalent on signaling proteins, transcription factors, metabolic enzymes, and histones and has emerged as a key regulator of diverse cellular processes, including signal transduction, gene expression, and protein degradation (7, 13–17).

This review focuses on the spatiotemporal regulation of key signaling pathways in glucose and lipid metabolism by *O*-GlcNAcylation. Understanding the regulatory role of this modification provides significant insight into normal liver physiology and liver disease processes.

## *O*-GlcNAc in Normal Liver Metabolism

### *O*-GlcNAc regulation of feeding response

**Figure 1:**
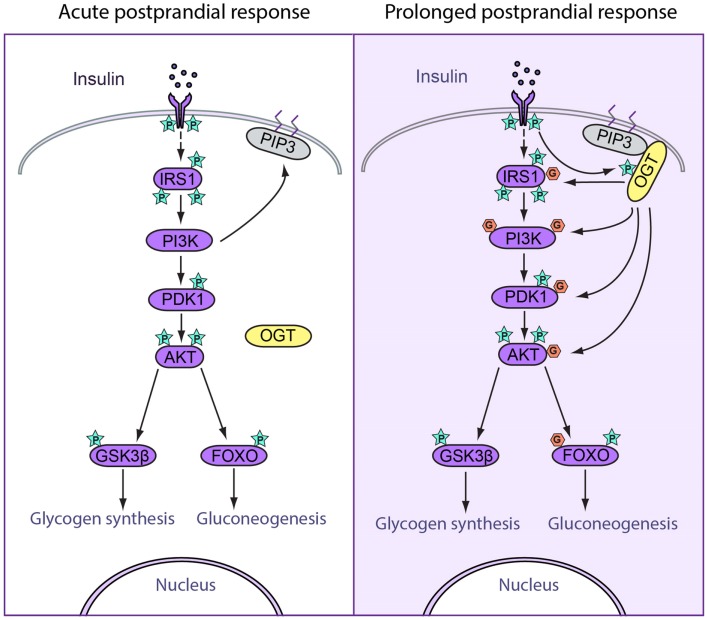
**Spatiotemporal regulation of feeding response by *O*-GlcNAcylation**. (Left) Acute postprandial response. During early insulin signaling, OGT remains in the cytosol. Insulin binds to insulin receptor (IR) and triggers its autophosphorylation. Phosphorylation of IR recruits IRS1 to be phosphorylated, after which IRS1 binds to PI3K. PI3K catalyzes the production of PIP3, which recruits PDK1 to be phosphorylated and activated. Activated PDK1 phosphorylates and activates AKT, which further phosphorylates and activated downstream targets, including GSK3β and FOXO, which enhances glycogen synthesis and suppresses gluconeogenesis. (Right) Prolonged postprandial response. The insulin signaling pathway needs to be attenuated after a period of stimulation in order to maintain homeostasis. *O*-GlcNAcylation of insulin signal proteins contributes to the attenuation of the pathway. During prolonged insulin signaling, OGT translocates to the plasma membrane and binds with PIP3 through the PIP3-binding domain. OGT is then phosphorylated and activated by IR. Activated OGT *O*-GlcNAcylates key insulin signaling proteins including IRS1, PI3K, PDK1, and AKT, antagonizing the activation by phosphorylation on these proteins. These events lead to decreased glycogen synthesis and increased gluconeogenesis.

Insulin plays a central role in glucose and lipid metabolism. After a meal, a sizable amount of insulin is rapidly released from the pancreas and is circulated to critical organs such as skeletal muscle, adipose tissue, and liver. In the liver, insulin induces acute activation of the insulin signaling cascade (acute postprandial response) followed by the attenuation of this pathway (prolonged postprandial response) (18). The temporal patterns of signal transduction are largely dictated by dynamic protein phosphorylation (19, 20). Similar to protein phosphorylation, *O*-GlcNAc modification can influence protein function by regulating protein–protein interaction, protein stability, nuclear-cytoplasmic shuttling, and intrinsic protein activity (21). The interplay between phosphorylation and *O*-GlcNAcylation has been implicated in the regulation of critical cellular processes. Here, we review the known effects of *O*-GlcNAc modification on insulin signal transduction in acute and prolonged postprandial responses, focusing on the role of *O*-GlcNAc in attenuating insulin signaling (Figure 1).

#### Acute postprandial response

In acute postprandial response, insulin binds to the insulin receptor (IR) and triggers the autophosphorylation of various tyrosine residues within the intracellular tyrosine kinase domain of the IR. This leads to the recruitment and phosphorylation of downstream proteins, including insulin receptor substrate (IRS). Subsequently, IRS binds and phosphorylates phosphatidylinositol-3-kinase (PI3K), which mediates a variety of critical signaling events. PI3K catalyzes the formation of membrane phosphatidylinositol 3,4,5-bisphosphate (PIP3), which recruits AKT to be activated by 3-phosphoinositide-dependent protein kinase 1 (PDK1) through phosphorylation at threonine 308. AKT then phosphorylates several target proteins, including glycogen synthase kinase (GSK3), AS160, and forkhead box protein O (FOXO). In glycogen synthesis, AKT phosphorylates and deactivates GSK3 (22), the enzyme responsible for phosphorylating and deactivating glycogen synthase. This leads to increased glycogen synthesis. In gluconeogenesis, phosphorylation of FOXO by AKT triggers FOXO export from the nucleus, thereby preventing FOXO from promoting gluconeogenic gene transcription (23). Thus, in acute insulin response, sequential phosphorylation events lead to increased glycogen synthesis and decreased gluconeogenic gene expression (Figure 1).

#### Prolonged postprandial response

The precise control of the duration of signal transduction is critical for maintaining physiological homeostasis. For instance, at some point after acute activation, insulin signal transduction is dampened through several feedback mechanisms (18). First, protein tyrosine phosphatases, such as PTP1B, have been shown to act as negative regulators of insulin signaling through dephosphorylation of IR (24). Lipid phosphatases, specifically PTEN and SHIP2, can dampen the PI3K pathway both *in vitro* and *in vivo* (25). Second, phosphorylation of specific Ser/Thr sites on IRS by protein kinases such as ribosomal protein S6 kinase beta-1 (S6K1) terminates insulin signaling (26, 27). Third, recent studies have indicated that *O*-GlcNAcylation plays a profound role in attenuating insulin signaling (28, 29).

In response to prolonged insulin stimulation, OGT translocates from the cytoplasm to the plasma membrane through the C-terminal PIP3-binding domain (28), leading to phosphorylation and activation of OGT by IR (30). Active OGT is known to *O*-GlcNAcylate and deactivate key insulin signaling proteins, including IRS-1, PI3K, PDK1, and AKT, thereby facilitating insulin signal attenuation (29, 30) (Figure 1).

Insulin receptor substrate deactivation is an important mechanism for terminating insulin signaling. IRS-1 is a direct substrate of OGT (29). Increased *O*-GlcNAcylation of IRS-1 in 3T3-L1 adipocytes reduces IRS-1 interaction with PI3K p85 and Tyr phosphorylation of IRS-1 at the Tyr 608 and increases IRS-1 phosphorylation at Ser 307 and Ser 632/635 (29). PI3K and PDK1 are also direct substrates of OGT and are implicated in insulin signaling attenuation (28).

Decreased AKT activity is essential for insulin signal termination. Increased *O*-GlcNAcylation of AKT at Thr 305/312 decreases AKT activity by reducing Thr 308 phosphorylation, which disrupts AKT/PDK1 interaction. In contrast, Ser 473 phosphorylation is unaffected (31).

Decreased AKT activity also reduces glycogen synthesis by decreasing phosphorylation of GSK3β. GSK3β is known to be modified by *O*-GlcNAcylation, and the inhibition of GSK3β by lithium alters global *O*-GlcNAc levels (32). However, the function of GSK3β *O*-GlcNAcylation has not yet been elucidated. Furthermore, *O*-GlcNAcylation of glycogen synthase itself is responsive to high glucose or glucosamine treatment and reduces the activity of the enzyme (33).

#### Lipogenesis in feeding response

Hepatic *de novo* lipogenesis allows for the conversion of glucose into fatty acids during feeding. Recent evidence indicates that glucose flux promotes lipogenesis through *O*-GlcNAcylation. The role of *O*-GlcNAcylation in both activating lipogenesis and attenuating insulin signaling raises an interesting question regarding the temporal regulation of insulin signaling. However, the studies on *O*-GlcNAcylation of lipogenic proteins have not addressed the dynamics of this modification in relation to the temporal regulation of lipogenesis.

The liver X receptors (LXRs) have long been viewed as nutrient sensors for lipid metabolism, glucose homeostasis, and inflammation. LXRs have been found to be *O*-GlcNAcylated in human Huh7 cells (34). High glucose increases LXR *O*-GlcNAcylation and transcriptional activity on the promoter of the sterol regulatory element-binding protein 1c (SREBP-1c), the master transcriptional regulator of hepatic lipogenesis (34). *In vivo* studies have shown that increased hepatic LXR *O*-GlcNAcylation can be observed in refed mice and in streptozotocin-induced diabetic mice (34).

The carbohydrate-responsive element-binding protein (ChRE BP) plays a significant role in glycolysis and lipogenesis. In HEK293T cells and hepatocytes, *O*-GlcNAcylation of ChREBP has been shown to stabilize the protein and to increase its transcriptional activity on lipogenic genes (35).

### *O*-GlcNAc regulation of fasting response

During fasting, energy metabolism shifts from glucose utilization to fat burning. In the liver, fasting induces glycogenolysis and gluconeogenesis in order to fuel glycolytic tissues, such as the brain and red blood cells. In short-term fasting, gluconeogenesis is mainly induced by glucagon through the cyclic AMP-CREB pathway. During a period of prolonged fasting, hepatic gluconeogenesis has shown to be sustained through the peroxisome proliferator-activated receptor γ co-activator 1α (PGC-1α)-dependent mechanisms (36).

#### Short-term fasting

During short-term fasting, glucagon stimulates gluconeogenesis by enhancing the activity of the cyclic AMP-responsive element-binding protein (CREB). CREB is phosphorylated at Ser 133 by cAMP-dependent Ser/Thr kinase protein kinase A (PKA) (37). Phosphorylation of CREB increases its interaction with CBP/p300 (38–40), which has been shown to promote gluconeogenic gene expression by acetylating nucleosomal histones (41–44). CREB directly enhances the expression of pyruvate carboxylase (PC), phosphoenolpyruvate carboxykinase 1 (PEPCK1), and glucose-6-phosphatase (G6PC) genes upon its binding to cAMP response elements (CREs). Phosphorylated CREB also promotes the expression of peroxisome proliferator-activated receptor-γ co-activator 1α (PGC1α), which is a critical co-activator for prolonged stimulation of gluconeogenic gene transcription (45).

**Figure 2:**
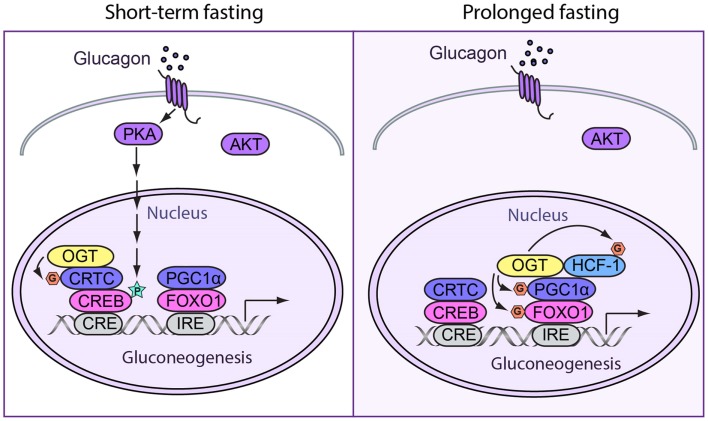
**Spatiotemporal regulation of fasting response by *O*-GlcNAcylation**. (Left) During short-term fasting, glucagon stimulates gluconeogenesis by enhancing the activity of CREB. Phosphorylation of CREB by PKA directly promotes gluconeogenic gene expression. Additionally, OGT can induce gluconeogenesis by *O*-GlcNAcylating CRTC2, the co-activator of CREB. When CRTC2 is dephosphorylated and then *O*-GlcNAcylated at the same site, it translocates into the nucleus and binds to CREB to induce gluconeogenesis. (Right) In prolonged fasting, OGT targets PGC-1α via a complex with HCF-1. Both PGC-1α and HCF-1 can be *O*-GlcNAcylated. *O*-GlcNAcylated PGC-1α helps recruit OGT to glycosylate and activate FOXO1, which further promotes hepatic glucose production.

*O*-GlcNAcylation of many gluconeogenic transcription factors and cofactors has been reported to promote glucose production in the liver. OGT can induce hepatic gluconeogenesis by *O*-GlcNAcylation of CRTC2, the co-activator of CREB. At basal levels, CRTCs are phosphorylated at Ser 70 and Ser 171 by salt-inducible kinases (SIKs) and other members of the AMP-activated protein kinase (AMPK) family and are sequestered in the cytoplasm by 14-3-3 proteins (46). In response to cAMP and calcium signals, CRTC2 is dephosphorylated and *O*-GlcNAcylated at the same site. This promotes CRTC2 translocation into the nucleus and binding to CREB, which induce gluconeogenesis (47) (Figure 2).

#### Prolonged fasting

During prolonged fasting, OGT primarily affects PGC1α-mediated expression of gluconeogenic genes. PGC1α acts as a co-activator for the glucocorticoid receptor, the hepatocyte nuclear factor 4 (HNF4), and FOXO1, which further stimulates the expression of gluconeogenic genes (45). OGT can target PGC-1α via host cell factor C1 (HCF-1) (8). *O*-GlcNAcylation stabilizes PGC-1α by recruiting BAP1 for de-ubiquitination (8). PGC-1α helps recruit OGT to *O*-GlcNAcylate and activate FOXO1 (48), which further promotes hepatic glucose production (Figure 2). Our previous work also demonstrates that OGT can physically and functionally interact with the glucocorticoid receptor. It is, therefore, plausible that OGT is also involved in glucocorticoid induction of gluconeogenesis (49).

In the above sections, we have provided a snapshot view of the molecular events regulated by *O*-GlcNAcylation in the different phases of the feeding/fasting cycle. It should be noted that these phases are not strictly divided but exist on the continuum of time. The feeding and fasting responses are directed by precise spatiotemporal regulation of insulin signaling cascades. Despite remarkable advances in our understanding of the role of *O*-GlcNAcylation in insulin signaling, how *O*-GlcNAcylation crosstalks with phosphorylation is not well known. Exploring the mechanistic and kinetic features of *O*-GlcNAcylation on key signaling proteins holds great promise for a better understanding of normal liver metabolism.

## *O*-GlcNAc in Liver Diseases

Non-alcoholic fatty liver disease is now the leading cause of liver disease in the US. NAFLD refers to a wide spectrum of liver disorders from simple steatosis (fatty liver) to NASH. One of the earliest features of NAFLD is accumulation of lipids in hepatocytes. A proportion of patients progress to NASH, which is characterized by ballooned hepatocytes, inflammatory infiltrate and fibrosis in the liver. Fatty liver disease is reported to be strongly associated with insulin resistance. Hepatic insulin resistance has a major impact on whole-body energy metabolism. Recent studies on *O*-GlcNAcylation shed light on the etiology of hepatic insulin resistance, fatty liver, and associated fibrosis.

### *O*-GlcNAc and hepatic insulin resistance

**Figure 3:**
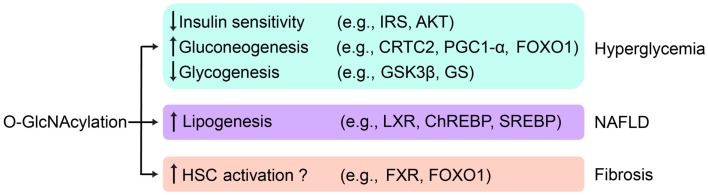
***O*-GlcNAc regulation of liver disease**. Aberrant hepatic *O*-GlcNAcylation leads to hyperglycemia by attenuating insulin signaling, activating gluconeogenesis, and suppressing glycogen synthesis. *O*-GlcNAcylation may contribute to NAFLD by stimulating *de novo* lipogenesis. *O*-GlcNAcylation may also play a role in the initiation and the progress of fibrosis by activating HSCs. Key targets of *O*-GlcNAcylation involved in related pathways are listed.

The liver is an insulin-sensitive organ critical for the maintenance of nutrient homeostasis. Hepatic insulin resistance produces derangements in liver metabolism such as uncontrolled glucose production, impaired glycogen synthesis, and enhanced lipogenesis. In the development of hepatic insulin resistance, *O*-GlcNAcylation is associated with various changes in gluconeogenesis, glycogenesis, and glycolysis. As discussed above, *O*-GlcNAcylation has been identified as a negative regulator of insulin signal transduction. Hepatic overexpression of OGT in mice impairs the expression of insulin-responsive genes and causes insulin resistance and dyslipidaemia (28, 50) (Figure 3).

Uncontrolled gluconeogenesis is one of the hallmarks of diabetic liver and contributes to hyperglycemia. *O*-GlcNAcylation has been found on many gluconeogenic transcription factors and cofactors, including CRTC2, HCF-1, PGC-1α, and FOXO1. Global *O*-GlcNAcylation levels have been shown to be elevated in the liver of high fat diet-fed and *db*/*db* mice. Hepatic overexpression of OGA in these mice decreases *O*-GlcNAcylation of CRTC2, downregulates gluconeogenic gene expression, and attenuates hyperglycemia (47). OGT *O-*GlcNAcylates and activates FOXO1 during prolonged fasting to stimulate gluconeogenesis. The levels of HCF-1 are elevated in the liver of high fat diet-fed and *db*/*db* mice, which is causally linked with uncontrolled gluconeogenesis and hyperglycemia. Consistently, knockdown of OGT and HCF-1 restores glucose homeostasis in *db*/*db* mice (8).

The liver undergoes glycogenesis to absorb excessive blood glucose. Glycogen synthase is activated when insulin signaling turns on and inhibits GSK3β. Activation of glycogen synthase is often suppressed in insulin resistance. High glucose has been shown to enhance *O*-GlcNAcylation of glycogen synthase, which is associated with reduced enzymatic activity in a cell culture model (33). This finding suggests that *O*-GlcNAcylation of glycogen synthase impairs glycogenesis and exacerbates hyperglycemia. This might produce a vicious cycle between hepatic insulin resistance and *O*-GlcNAcylation.

Recent studies also indicate that *O*-GlcNAcylation modulates glycolysis by inhibiting phosphofructokinase 1 (PFK1) activity and redirecting glucose flux into the pentose phosphate pathway (PPP) (17). Overexpression of OGT or pharmacological inhibition of OGA in many cell lines leads to increased global *O*-GlcNAcylation, decreased glycolysis, and decreased ATP concentration. Further studies should clarify whether *O*-GlcNAcylation inhibits glycolysis in the liver and whether this contributes to the pathogenesis of insulin resistance.

### *O*-GlcNAc and NAFLD

Non-alcoholic fatty liver disease is characterized by triglyceride accumulation in the cytoplasm of hepatocytes, arising from an imbalance between lipid acquisition and removal. Insulin insensitivity presumably leads to suppressed lipogenesis, which may alleviate NAFLD symptoms. However, diabetic animals often have “selective insulin resistance,” where insulin fails to suppress gluconeogenesis but retains its ability to activate lipogenesis (51). The conundrum of selective insulin resistance has not been resolved. It is also under debate whether selective insulin resistance and NAFLD are inherently related to each other.

The role of *O*-GlcNAcylation in regulating lipogenesis might hold the key to this paradox because *O*-GlcNAcylation suppresses insulin signaling but activates lipogenic pathways. *O*-GlcNAcylation increases ChREBP protein level and transcriptional activity on lipogenic genes (35). Importantly, ChREBP is hyper-*O*-GlcNAcylated in the liver of *db*/*db* mice. OGA overexpression reduces ChREBP glycosylation and protects these mice from hepatic steatosis. Another paradox involves farnesoid X receptor (FXR), a nuclear receptor that inhibits expression of SREBP1c and LXRα (52). Patients with NAFLD have lower levels of FXR mRNA and protein (53). An independent study has demonstrated that high glucose concentrations, which are believed to be a common pathophysiological condition in NAFLD patients, increases FXR *O*-GlcNAcylation and enhances FXR gene expression and protein stability (54). More direct evidence is required to reveal how *O*-GlcNAcylation affects NAFLD progression through FXR (Figure 3).

### *O*-GlcNAc and liver fibrosis

As NAFLD progresses to NASH, fibrosis becomes one of the common features among NASH patients and often correlates with poor prognosis. Fibrosis is characterized by excessive deposition of the extracellular matrix and can be regarded as a scarring process of the liver in response to repeated injury. NASH patients with liver fibrosis are more susceptible to cirrhosis (4), which is believed to be more irreversible than fibrosis. Therefore, fibrosis serves as a valuable therapeutic target to retard the progression of NASH. To date, the evidence that links *O*-GlcNAcylation to fibrosis is still limited but does shed some light on the topic.

Activated hepatic stellate cells (HSCs) are the major source of the extracellular matrix in the liver (55). It was reported that elevated levels of *O*-GlcNAcylation are essential for HSC activation and upregulation of collagen expression *in vitro* (56). Both *in vitro* and *in vivo* studies confirm that FXR activation limits the transdifferentiation of HSCs from a resting, fat-storing phenotype toward a myofibroblast-like phenotype (57, 58). Given their effects on lowering inflammatory and fibrogenic processes, a number of synthetic FXR agonists are being used to treat different hepatic and metabolic disorders (59, 60). Nevertheless, the role of FXR *O*-GlcNAcylation in the context of fibrosis has not been elucidated. FOXO, which can be *O*-GlcNAcylated, also has a potential role in fibrosis. A study showed that HSC transdifferentiation was suppressed by FOXO1 (61). Paradoxically, enhanced FOXO1 expression and nuclear localization were reported in NASH patients (62). Further study is required to address whether *O*-GlcNAcylation of FOXO1 plays a role in liver fibrosis (Figure 3).

## Conclusion

It is becoming clear that protein *O*-GlcNAcylation is critical for metabolic control in time and space. In hepatocytes, cytosolic *O*-GlcNAc is crucially involved in resetting insulin signaling, whereas nuclear *O*-GlcNAc has a key role in transcriptional regulation of gluconeogenesis and lipogenesis. This regulatory mechanism may serve as a “rheostat” that ensures the fluctuation of circulating nutrients within a limited range during the feeding/fasting cycle. Under pathophysiological conditions such as overnutrition or stress, aberrant cellular *O*-GlcNAcylation leads to excessive glucose production and lipid accumulation in the liver. As such, *O*-GlcNAc disturbance is likely a unifying cause of hyperglycemia, fatty liver, and fibrosis. Small molecules that target *O*-GlcNAc signaling should be explored to treat these medical conditions.

## Conflict of Interest Statement

The authors declare that the research was conducted in the absence of any commercial or financial relationships that could be construed as a potential conflict of interest.

## References

[1] Chalasani N Younossi Z Lavine JE Diehl AM Brunt EM Cusi K The diagnosis and management of non-alcoholic fatty liver disease: Practice Guideline by the American Association for the Study of Liver Diseases, American College of Gastroenterology, and the American Gastroenterological Association. Hepatology (2012) 55(6):2005–23 10.1002/hep.25762 22488764

[2] Tolman KG Fonseca V Dalpiaz A Tan MH Spectrum of liver disease in type 2 diabetes and management of patients with diabetes and liver disease. Diabetes Care (2007) 30(3):734–43 10.2337/dc06-1539 17327353

[3] Kleiner DE Brunt EM Van Natta M Behling C Contos MJ Cummings OW Design and validation of a histological scoring system for nonalcoholic fatty liver disease. Hepatology (2005) 41(6):1313–21. 10.1002/hep.20701 15915461

[4] Hui JM Kench JG Chitturi S Sud A Farrell GC Byth K Long-term outcomes of cirrhosis in nonalcoholic steatohepatitis compared with hepatitis C. Hepatology (2003) 38(2):420–7. 10.1053/jhep.2003.50320 12883486

[5] Hart GW Slawson C Ramirez-Correa G Lagerlof O. Cross talk between *O*-GlcNAcylation and phosphorylation: roles in signaling, transcription, and chronic disease. Annu Rev Biochem (2011) 80:825. 10.1146/annurev-biochem-060608-102511 21391816 PMC3294376

[6] Ruan HB Singh JP Li MD Wu J Yang X. Cracking the *O-*GlcNAc code in metabolism. Trends Endocrinol Metab (2013) 24(6):301–9. 10.1016/j.tem.2013.02.002 23647930 PMC3783028

[7] Hardiville S Hart GW. Nutrient regulation of signaling, transcription, and cell physiology by *O-*GlcNAcylation. Cell Metab (2014) 20(2):208–13. 10.1016/j.cmet.2014.07.014 25100062 PMC4159757

[8] Ruan HB Han X Li MD Singh JP Qian K Azarhoush S *O-*GlcNAc transferase/host cell factor C1 complex regulates gluconeogenesis by modulating PGC-1α stability. Cell Metab (2012) 16(2):226–37. 10.1016/j.cmet.2012.07.006 22883232 PMC3480732

[9] Ruan HB Dietrich MO Liu ZW Zimmer MR Li MD Singh JP *O-*GlcNAc transferase enables AgRP neurons to suppress browning of white fat. Cell (2014) 159(2):306–17. 10.1016/j.cell.2014.09.010 25303527 PMC4509746

[10] Janetzko J Walker S. The making of a sweet modification: structure and function of *O-*GlcNAc transferase. J Biol Chem (2014). 10.1074/jbc.R114.604405 25336649 PMC4263849

[11] Vocadlo DJ. *O-*GlcNAc processing enzymes: catalytic mechanisms, substrate specificity, and enzyme regulation. Curr Opin Chem Biol (2012) 16(5–6):488–97. 10.1016/j.cbpa.2012.10.021 23146438

[12] Alonso J Schimpl M van Aalten DM. *O-*GlcNAcase: promiscuous hexosaminidase or key regulator of *O-*GlcNAc signalling? J Biol Chem (2014). 10.1074/jbc.R114.609198 25336650 PMC4263850

[13] Singh JP Zhang K Wu J Yang X *O-*GlcNAc signaling in cancer metabolism and epigenetics. Cancer Lett (2015) 356(2):244–50 10.1016/j.canlet.2014.04.014 24769077 PMC4208982

[14] Ruan HB Nie Y Yang X. Regulation of protein degradation by *O-*GlcNAcylation: crosstalk with ubiquitination. Mol Cell Proteomics (2013) 12(12):3489–97. 10.1074/mcp.R113.029751 23824911 PMC3861702

[15] Hanover JA Krause MW Love DC. Bittersweet memories: linking metabolism to epigenetics through *O-*GlcNAcylation. Nat Rev Mol Cell Biol (2012) 13(5):312–21. 10.1038/nrm3334 22522719

[16] Li MD Ruan HB Hughes ME Lee JS Singh JP Jones SP *O-*GlcNAc signaling entrains the circadian clock by inhibiting BMAL1/CLOCK ubiquitination. Cell Metab (2013) 17(2):303–10. 10.1016/j.cmet.2012.12.015 23395176 PMC3647362

[17] Yi W Clark PM Mason DE Keenan MC Hill C Goddard WA 3rd Phosphofructokinase 1 glycosylation regulates cell growth and metabolism. Science (2012) 337(6097):975–80. 10.1126/science.1222278 22923583 PMC3534962

[18] Saltiel AR Pessin JE Insulin signaling pathways in time and space. Trends Cell Biol (2002) 12(2):65–71 10.1016/S0962-8924(01)02207-3 11849969

[19] Kubota H Noguchi R Toyoshima Y Ozaki Y Uda S Watanabe K Temporal coding of insulin action through multiplexing of the AKT pathway. Mol Cell (2012) 46(6):820–32. 10.1016/j.molcel.2012.04.018 22633957

[20] Purvis JE Lahav G. Decoding the insulin signal. Mol Cell (2012) 46(6):715–6. 10.1016/j.molcel.2012.06.005 22749395 PMC4142421

[21] Ozcan S Andrali SS Cantrell JE. Modulation of transcription factor function by *O-*GlcNAc modification. Biochim Biophys Acta (2010) 1799(5–6):353–64. 10.1016/j.bbagrm.2010.02.005 20202486 PMC2881704

[22] Fang X Yu SX Lu Y Bast RC Jr Woodgett JR Mills GB. Phosphorylation and inactivation of glycogen synthase kinase 3 by protein kinase A. Proc Natl Acad Sci U S A (2000) 97(22):11960–5. 10.1073/pnas.220413597 11035810 PMC17277

[23] Manning BD Cantley LC. AKT/PKB signaling: navigating downstream. Cell (2007) 129(7):1261–74. 10.1016/j.cell.2007.06.009 17604717 PMC2756685

[24] Asante-Appiah E Kennedy BP. Protein tyrosine phosphatases: the quest for negative regulators of insulin action. Am J Physiol Endocrinol Metab (2003) 284(4):E663–70. 10.1152/ajpendo.00462.2002 12626322

[25] Lazar DF Saltiel AR. Lipid phosphatases as drug discovery targets for type 2 diabetes. Nat Rev Drug Discov (2006) 5(4):333–42. 10.1038/nrd2007 16582877

[26] Zick Y. Ser/Thr phosphorylation of IRS proteins: a molecular basis for insulin resistance. Sci STKE (2005) 2005(268):e4. 10.1126/stke.2682005pe4 15671481

[27] Zhang HH Lipovsky AI Dibble CC Sahin M Manning BD. S6K1 regulates GSK3 under conditions of mTOR-dependent feedback inhibition of Akt. Mol Cell (2006) 24(2):185–97. 10.1016/j.molcel.2006.09.019 17052453 PMC1880887

[28] Yang X Ongusaha PP Miles PD Havstad JC Zhang F So WV Phosphoinositide signalling links *O-*GlcNAc transferase to insulin resistance. Nature (2008) 451(7181):964–9. 10.1038/nature06668 18288188

[29] Whelan SA Dias WB Thiruneelakantapillai L Lane MD Hart GW. Regulation of insulin receptor substrate 1 (IRS-1)/AKT kinase-mediated insulin signaling by O-Linked beta-*N-*acetylglucosamine in 3T3-L1 adipocytes. J Biol Chem (2010) 285(8):5204–11. 10.1074/jbc.M109.077818 20018868 PMC2820748

[30] Whelan SA Lane MD Hart GW. Regulation of the O-linked beta-*N-*acetylglucosamine transferase by insulin signaling. J Biol Chem (2008) 283(31):21411–7. 10.1074/jbc.M800677200 18519567 PMC2490780

[31] Wang S Huang X Sun D Xin X Pan Q Peng S Extensive crosstalk between *O-*GlcNAcylation and phosphorylation regulates Akt signaling. PLoS One (2012) 7(5):e37427. 10.1371/journal.pone.0037427 22629392 PMC3358304

[32] Wang Z Pandey A Hart GW. Dynamic interplay between O-linked *N-*acetylglucosaminylation and glycogen synthase kinase-3-dependent phosphorylation. Mol Cell Proteomics (2007) 6(8):1365–79. 10.1074/mcp.M600453-MCP200 17507370

[33] Parker GJ Lund KC Taylor RP McClain DA. Insulin resistance of glycogen synthase mediated byo-linked *N-*acetylglucosamine. J Biol Chem (2003) 278(12):10022–7. 10.1074/jbc.M207787200 12510058

[34] Anthonisen EH Berven L Holm S Nygård M Nebb HI Grønning-Wang LM. Nuclear receptor liver X receptor is *O-*GlcNAc-modified in response to glucose. J Biol Chem (2010) 285(3):1607–15. 10.1074/jbc.M109.082685 19933273 PMC2804318

[35] Guinez C Filhoulaud G Rayah-Benhamed F Marmier S Dubuquoy C Dentin R *O-*GlcNAcylation increases ChREBP protein content and transcriptional activity in the liver. Diabetes (2011) 60(5):1399–413. 10.2337/db10-0452 21471514 PMC3292313

[36] Altarejos JY Montminy M. CREB and the CRTC co-activators: sensors for hormonal and metabolic signals. Nat Rev Mol Cell Biol (2011) 12(3):141–51. 10.1038/nrm3072 21346730 PMC4324555

[37] Gonzalez GA Montminy MR. Cyclic AMP stimulates somatostatin gene transcription by phosphorylation of CREB at serine 133. Cell (1989) 59(4):675–80. 10.1016/0092-8674(89)90013-5 2573431

[38] Goodman RH Smolik S CBP/p300 in cell growth, transformation, and development. Genes Dev (2000) 14(13):1553–77 10.1101/gad.14.13.1553 10887150

[39] Lundblad JR Kwok RP Laurance ME Harter ML Goodman RH. Adenoviral E1A-associated protein p300 as a functional homologue of the transcriptional co-activator CBP. Nature (1995) 374(6517):85–8. 10.1038/374085a0 7870179

[40] Parker D Ferreri K Nakajima T LaMorte VJ Evans R Koerber SC Phosphorylation of CREB at Ser-133 induces complex formation with CREB-binding protein via a direct mechanism. Mol Cell Biol (1996) 16(2):694–703. 8552098 10.1128/mcb.16.2.694 PMC231049

[41] Michael LF Asahara H Shulman AI Kraus WL Montminy M. The phosphorylation status of a cyclic AMP-responsive activator is modulated via a chromatin-dependent mechanism. Mol Cell Biol (2000) 20(5):1596–603. 10.1128/MCB.20.5.1596-1603.2000 10669737 PMC85343

[42] Bannister AJ Kouzarides T. The CBP co-activator is a histone acetyltransferase. Nature (1996) 384(6610):641–3. 10.1038/384641a0 8967953

[43] Ogryzko VV Schiltz RL Russanova V Howard BH Nakatani Y. The transcriptional coactivators p300 and CBP are histone acetyltransferases. Cell (1996) 87(5):953–9. 10.1016/S0092-8674(00)82001-2 8945521

[44] Asahara H Santoso B Guzman E Du K Cole PA Davidson I Chromatin-dependent cooperativity between constitutive and inducible activation domains in CREB. Mol Cell Biol (2001) 21(23):7892–900. 10.1128/MCB.21.23.7892-7900.2001 11689682 PMC99956

[45] Herzig S Long F Jhala US Hedrick S Quinn R Bauer A CREB regulates hepatic gluconeogenesis through the coactivator PGC-1. Nature (2001) 413(6852):179–83. 10.1038/35098123 11557984

[46] Uebi T Tamura M Horike N Hashimoto YK Takemori H. Phosphorylation of the CREB-specific coactivator TORC2 at Ser(307) regulates its intracellular localization in COS-7 cells and in the mouse liver. Am J Physiol Endocrinol Metab (2010) 299(3):E413–25. 10.1152/ajpendo.00525.2009 20551288

[47] Dentin R Hedrick S Xie J Yates J 3rd Montminy M. Hepatic glucose sensing via the CREB coactivator CRTC2. Science (2008) 319(5868):1402–5. 10.1126/science.1151363 18323454

[48] Housley MP Udeshi ND Rodgers JT Shabanowitz J Puigserver P Hunt DF A PGC-1alpha-*O-*GlcNAc transferase complex regulates FoxO transcription factor activity in response to glucose. J Biol Chem (2009) 284(8):5148–57. 10.1074/jbc.M808890200 19103600 PMC2643526

[49] Li MD Ruan HB Singh JP Zhao L Zhao T Azarhoush S *O-*GlcNAc transferase is involved in glucocorticoid receptor-mediated transrepression. J Biol Chem (2012) 287(16):12904–12. 10.1074/jbc.M111.303792 22371499 PMC3339970

[50] Dentin R Liu Y Koo SH Hedrick S Vargas T Heredia J Insulin modulates gluconeogenesis by inhibition of the coactivator TORC2. Nature (2007) 449(7160):366–9. 10.1038/nature06128 17805301

[51] Brown MS Goldstein JL. Selective versus total insulin resistance: a pathogenic paradox. Cell Metab (2008) 7(2):95–6. 10.1016/j.cmet.2007.12.009 18249166

[52] Watanabe M Houten SM Wang L Moschetta A Mangelsdorf DJ Heyman RA Bile acids lower triglyceride levels via a pathway involving FXR, SHP, and SREBP-1c. J Clin Invest (2004) 113(10):1408–18. 10.1172/JCI21025 15146238 PMC406532

[53] Yang Z-X Shen W Sun H. Effects of nuclear receptor FXR on the regulation of liver lipid metabolism in patients with non-alcoholic fatty liver disease. Hepatol Int (2010) 4(4):741–8. 10.1007/s12072-010-9202-6 21286345 PMC2994619

[54] Berrabah W Aumercier P Gheeraert C Dehondt H Bouchaert E Alexandre J Glucose sensing *O-*GlcNAcylation pathway regulates the nuclear bile acid receptor farnesoid X receptor (FXR). Hepatology (2014) 59(5):2022–33. 10.1002/hep.26710 24037988

[55] Pinzani M Rombouts K. Liver fibrosis: from the bench to clinical targets. Dig Liver Dis (2004) 36(4):231–42. 10.1016/j.dld.2004.06.001 15115333

[56] Fan X Chuan S Hongshan W. Protein O glycosylation regulates activation of hepatic stellate cells. Inflammation (2013) 36(6):1248–52. 10.1007/s10753-013-9662-7 23743764

[57] Fujino T Une M Imanaka T Inoue K Nishimaki-Mogami T. Structure-activity relationship of bile acids and bile acid analogs in regard to FXR activation. J Lipid Res (2004) 45(1):132–8. 10.1194/jlr.M300215-JLR200 13130122

[58] Fiorucci S Antonelli E Rizzo G Renga B Mencarelli A Riccardi L The nuclear receptor SHP mediates inhibition of hepatic stellate cells by FXR and protects against liver fibrosis. Gastroenterology (2004) 127(5):1497–512. 10.1053/j.gastro.2004.08.001 15521018

[59] Downes M Verdecia MA Roecker AJ Hughes R Hogenesch JB Kast-Woelbern HR A chemical, genetic, and structural analysis of the nuclear bile acid receptor FXR. Mol Cell (2003) 11(4):1079–92. 10.1016/S1097-2765(03)00104-7 12718892 PMC6179153

[60] Thomas C Pellicciari R Pruzanski M Auwerx J Schoonjans K. Targeting bile-acid signalling for metabolic diseases. Nat Rev Drug Discov (2008) 7(8):678–93. 10.1038/nrd2619 18670431

[61] Adachi M Osawa Y Uchinami H Kitamura T Accili D Brenner DA. The forkhead transcription factor FoxO1 regulates proliferation and transdifferentiation of hepatic stellate cells. Gastroenterology (2007) 132(4):1434–46. 10.1053/j.gastro.2007.01.033 17408630

[62] Valenti L Rametta R Dongiovanni P Maggioni M Fracanzani AL Zappa M Increased expression and activity of the transcription factor FOXO1 in nonalcoholic steatohepatitis. Diabetes (2008) 57(5):1355–62. 10.2337/db07-0714 18316359

